# Integrative omics analysis reveals epigenomic and transcriptomic signatures underlying brain structural deficits in major depressive disorder

**DOI:** 10.1038/s41398-023-02724-8

**Published:** 2024-01-09

**Authors:** Junjie Zheng, Fay Y. Womer, Lili Tang, Huiling Guo, Xizhe Zhang, Yanqing Tang, Fei Wang

**Affiliations:** 1 Early Intervention Unit, Department of Psychiatry, The Affiliated Brain Hospital of Nanjing Medical University, Nanjing, China; 2 Functional Brain Imaging Institute of Nanjing Medical University, Nanjing, China; 3 Department of Psychiatry and Behavioral Sciences, Vanderbilt University Medical Center, Nashville, TN USA; 4 School of Biomedical Engineering and Informatics, Nanjing Medical University, Nanjing, China; 5 Department of Psychiatry, The First Hospital of China Medical University, Shenyang, China; 6 Brain Function Research Section, The First Hospital of China Medical University, Shenyang, China; 7 Department of Gerontology, The First Hospital of China Medical University, Shenyang, China; 8 Shengjing Hospital of China Medical University, Shenyang, China; 9 Department of Mental Health, School of Public Health, Nanjing Medical University, Nanjing, China

**Keywords:** Depression, Epigenetics in the nervous system

## Abstract

Several lines of evidence support the involvement of transcriptomic and epigenetic mechanisms in the brain structural deficits of major depressive disorder (MDD) separately. However, research in these two areas has remained isolated. In this study, we proposed an integrative strategy that combined neuroimaging, brain-wide gene expression, and peripheral DNA methylation data to investigate the genetic basis of gray matter abnormalities in MDD. The MRI T1-weighted images and Illumina 850 K DNA methylation microarrays were obtained from 269 patients and 416 healthy controls, and brain-wide transcriptomic data were collected from Allen Human Brain Atlas. The between-group differences in gray matter volume (GMV) and differentially methylated CpG positions (DMPs) were examined. The genes with their expression patterns spatially related to GMV changes and genes with DMPs were overlapped and selected. Using principal component regression, the associations between DMPs in overlapped genes and GMV across individual patients were investigated, and the region-specific correlations between methylation status and gene expression were examined. We found significant associations between the decreased GMV and DMPs methylation status in the anterior cingulate cortex, inferior frontal cortex, and fusiform face cortex regions. These DMPs genes were primarily enriched in the neurodevelopmental and synaptic transmission process. There was a significant negative correlation between DNA methylation and gene expression in genes associated with GMV changes of the frontal cortex in MDD. Our findings suggest that GMV abnormalities in MDD may have a transcriptomic and epigenetic basis. This imaging-transcriptomic-epigenetic integrative analysis provides spatial and biological links between cortical morphological deficits and peripheral epigenetic signatures in MDD.

## Introduction

Altered gray matter volumes (GMV) in key cognitive and emotional brain regions, such as the medial and inferior prefrontal cortex (MPFC, IFC), the dorsolateral prefrontal cortex (DLPFC), and the anterior cingulate cortex (ACC) have consistently been implicated in major depressive disorder (MDD) [1–4]. Genetic and environmental factors play a significant role in shaping gray matter (GM) structures and contribute to the GM abnormalities in MDD [5, 6]. The regional GM abnormalities of the cerebral cortex are highly heritable and result from a complex genetic architecture involving multiple biological processes [7]. Genome-wide association studies (GWAS) have identified hundreds of genomic loci as genetic susceptibility factors in the pathogenesis of MDD [8]. Risk genotypes appear to be associated with diminished neurotransmitter uptake at synaptic terminals, resulting in decreased GMV in brain regions involved in emotion processing, particularly the prefrontal cortex and cingulate cortex in MDD [9, 10]. Genotype is not the sole determinant of phenotypes in the disorder [8]. In fact, genetic variation appears to explain only a small proportion of GMV alterations in MDD [11]. Risk genes may contribute to GMV reduction through their effects on gene expression, which more directly reflect the genetic processes responsible for GM structure [12, 13]. Further, epigenetic processes, such as DNA methylation, influence if and how genes are expressed and may mediate the effects of gene-gene or gene-environment interactions in MDD [11, 14]. They are also considered major mechanisms for neural plasticity [11, 15]. There is converging evidence to suggest that epigenetic effects contribute to GM atrophy in prefrontal cortex regions in MDD [16, 17]. Variations in DNA methylation have been shown to differentially correlate with cortical thickness in frontal, temporal, parietal, and occipital brain regions in MDD [18]. Because MDD arises from a complex genetic landscape involving multiple genes, previous neuroimaging studies involving DNA methylation of specific disease mechanisms may have limited relevance to the disorder [5, 19, 20]. A more comprehensive understanding of the epigenetic effects can be obtained through epigenomic studies. Therefore, integrating transcriptomic, epigenomic, and neuroimaging data may provide a more comprehensive in-vivo perspective of genetic influences on GMV alterations in MDD.

The development of gene expression brain atlases and molecular arrays has significantly advanced the field of imaging transcriptomics in recent years [12]. The integration of transcriptomic and neuroimaging data provided by these atlases offers a more comprehensive framework for genomic and whole-brain analyses and allows for the testing of gene-brain region hypotheses and potential mechanisms in mental illness [21–23]. The publicly accessible Allen Human Brain Atlas (AHBA) maps regional gene expression from postmortem samples across a healthy brain, providing a spatial brain atlas of gene expression at the level of mRNA transcription [12]. The AHBA has been used in MDD studies by integrating the brain-wide transcriptomic microarray and statistical brain mappings [21, 24, 25]. A recent study found that neuronal-specific transcriptional changes, which are involved in synaptic transmission and major monoamine neurotransmitter systems, account for cortical structural differences in patients with MDD compared to healthy controls [26–28]. Another recent meta-analysis found that GMV changes in MDD were linked with mRNA expression of genes involved in neuronal development, metabolism, immune response, and transmembrane transport [28]. It is important to note that this meta-analysis only utilized coordinates from published studies and did not include individual subject data. This could lead to limitations and inaccuracies in the results [29]. Notably, imaging transcriptomic analysis is a framework to identify spatial correlation patterns across regions but not across individuals [21, 25, 26]. Moreover, it is also necessary to understand how such general transcriptional correlates of neuroimaging variations represent region-specific molecular underpinnings of brain structural alterations [28]. Identifying region-specific molecular mechanisms underlying GMV alterations in MDD may have important clinical implications in developing novel imaging phenotype guidance for precision medicine approaches [30].

The integration of DNA methylation arrays with gene expression brain atlases like the AHBA provides valuable insights into the role of epigenomics in the regulation of gene expression and its effects on GMV alterations in MDD. Additionally, the moderate correlation between peripheral DNA methylation and brain DNA methylation indicates that peripheral DNA methylation could be used as a proxy for brain DNA methylation, providing a more accessible and cost-effective method for epigenomic analysis [11, 31, 32]. Converging evidence suggests that epigenetic changes are associated with gray matter atrophy in the key brain regions of MDD [16, 17, 33]. The traditional research is based on single or couples of genes to analyze the correlation between DNA methylation and brain structural changes in MDD [5, 19, 20]. Few studies have integrated the associations among brain imaging phenotypes, gene expression, and DNA methylation at the genome-wide level, which can bring together multi-omics data to offer a more complete understanding of how molecular alterations at the microscale level contribute to macroscale brain abnormalities in MDD.

In this study, we aim to shed light on the molecular underpinnings of GMV alterations in MDD by conducting a comprehensive analysis. First, we compare GMV between patients with MDD and HC. Next, we use the AHBA to identify genes whose mRNA expression is spatially correlated with GMV changes. Subsequently, we utilize DNA methylation arrays to identify differentially methylated positions (DMPs) in MDD compared to HC. We then focus on DMPs within the biological pathways enriched for genes that are related to GMV changes. Finally, we explore the links between multiple DNA methylation changes and GMV alterations in individual patients with MDD. To gain further insight, we analyze the relationship between DNA methylation and gene expression across genes to better understand how epigenetic modifications impact GMV in the disorder. Based on the findings from the imaging transcriptomic and imaging epigenetic studies mentioned above, we hypothesized that GMV changes in MDD may have a transcriptomic and epigenetic basis.

## Materials and methods

### Participants

We recruited 269 patients with MDD and 458 healthy controls (HC). The patients with MDD were recruited from the inpatient department of the Shenyang Mental Health Center and the outpatient clinic of the Department of Psychiatry of the First Affiliated Hospital of China Medical University in Shenyang, China. Participants over 18 years old provided written consent themselves. For those under 18, a parent or legal guardian provided written informed consent. This study was approved by the Ethics Committee of the first affiliated Hospital of China Medical University. Details about all participants’ demographic and clinical information, including diagnostic procedures and clinical ethics, are presented in the Supplementary Materials.

### Image acquisition and MRI processing

The details of Structural MRI scanning parameters and acquisition requirements for participants are presented in Supplementary Materials T1-weighted images were preprocessed using the Computation Anatomy Toolbox (CAT 12; Christian Gaser; Department of Psychiatry, University of Jena) [34] implemented in Statistical Parametric Mapping (SPM 12; Wellcome Department of Cognitive Neurology, University of London, UK) for voxel-based morphometry(VBM). The HCP (https://humanconnectome.org/) atlas was used as the template [35]. This parcellation was used to extract mean values of regional gray matter volume density within all ROIs as ROI-wise GMV for each subject, which were used in previous studies [36, 37]. ROI-wise, GMV was used to investigate abnormalities in each brain region in MDD. Details are presented in the Supplementary Materials.

### GMV comparison between groups

**Fig. 1:**
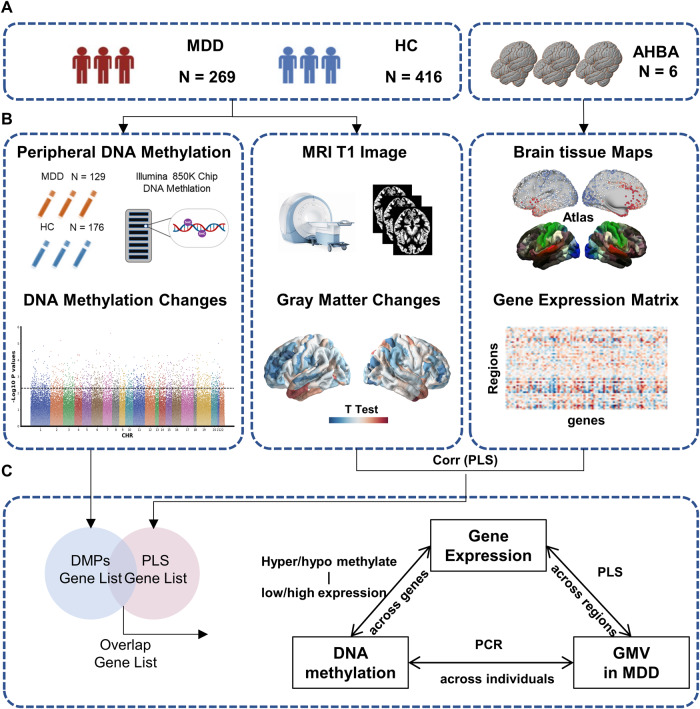
Schematic overview of the workflow in this study. **a** The multi-omics data were from 269 patients with MDD, 416 healthy controls, and 6 postmortem donors from AHBA(http://human.brain-map.org). **b** Peripheral DNA methylation was tested by illumine 850 K chip from 129 MDD and 176 HC, and abnormal CpG sites in the promoter region were identified in MDD compared to HC; T1 images and gray matter volume were collected from all participants; the gray matter volume changes in MDD were tested; Brainwide gene expression was coregistered to HCP atlas to produce a 180(regions) × 10,027(genes) gene expression matrix. **c** Partial least squares (PLS) regression was then used to identify imaging transcriptomic associations. The DNA methylation of CpG sites in overlaps between imaging transcriptomic associated genes and methylated changes genes were selected. Finally, principle component analysis (PCA) features reduction and stepwise multiple linear regression (SMLR) were performed to find the associations between regional GMV changes and DNA methylation changes across individuals with MDD. We also test the relationships between epigenetic changes and gene expressions across GMV-related genes.

To identify GMV abnormalities in MDD, we performed a general linear model (GLM) and *t*-test between MDD and HC groups for each brain region, with age, gender, education, medication status, and total intracranial volume (TIV) as covariates. Medication status was included as a covariate since 131 MDD participants (48% of the MDD group) were taking antidepressants, and 8 patients (0.02% of the MDD group) were taking antipsychotic drugs. Abnormal GMV regions were selected using a significance level of *p* < 0.05, FDR corrected. An overview of the study is presented in Fig. 1.

### Imaging transcriptomic analysis using GMV changes and gene expression

Transcriptional profiles were obtained from 3702 brain tissue samples of 6 donors from the Allen Human Brain Atlas (AHBA) website (https://human.brain-map.org/). The expression data underwent preprocessing following five previously reported major steps [38], with further details provided in the Supplementary Methods. The examination of transcriptome-neuroimaging relationships across different groups was limited by the use of gene expression profiles from only six postmortem healthy human brains in the AHBA. Additionally, the AHBA dataset included data for the right hemisphere of the brain for only two participants, which restricts the representation of the entire brain in relation to the transcriptional changes and MDD-related GMV alterations. Using partial least squares (PLS) regression, as previously performed in other imaging transcriptomics studies [24, 26], we examined the spatial associations between the T-statistic map of GMV alterations and gene expression values in the HCP Atlas left hemisphere (180 regions). Then, we selected the first component in the PLS regression (PLS1) genes with weights |*Z*| > 3, *p* < 0.05 corrected as candidate genes whose spatial expression correlated with GMV abnormalities in MDD. The details were provided in the Supplementary Materials.

### Principal component regression between individual GMV and DNA methylation

The Infinium Human Methylation850 (850 K) microarray was used to assess DNA methylation levels. The DNA methylation levels were expressed as *β*-values at the targeted CpG site. We presented the details of DNA methylation preprocessing in the Supplementary Methods. we performed a general linear model (GLM) and t-test for each CpG site with age, gender, education, and medication as covariates to identify differentially methylated CpG positions (DMPs) in MDD compared to HC. We then selected the DMPs of genes as features from the pathways in which the overlapped genes between PLS1 genes and DMPs genes enriched. The feature selection processing details were described in the Supplementary Methods.

We used principal component regression (PCR), which combined principal component analysis (PCA) and linear regression to examine the correlation between regional GMV and DMPs across all patients with MDD. Then, PCA was calculated to reduce the high dimensions of DMPs features. The top components that explained up to 80% of the accumulated variance were selected. Then, we used a stepwise multiple linear regression (SMLR) model to test the associations between GMV changes and DMPs components within the MDD group. The significant level of the SMLR models was set at *p* < 0.05, and FDR correction was used to control the false positive rate. We also performed leave one out cross validation (LOOCV) to validate DMPs features prediction for GMV in patients with MDD. The details of the LOOCV processing were presented in the Supplementary Materials. We selected top-weighted DMPs in GMV-related DMPs components and used Spearman linear correlation to calculate the associations between DMPs methylated states and gene expression of their annotated genes in each GMV-changed region. The details were presented in the Supplementary Materials. We also validated the consistent methylation status between blood and brain in the Top DMPs using online tools (blood–brain DNA methylation comparison tool, https://epigenetics.essex.ac.uk/bloodbrain/, and blood–brain epigenetic concordance; BECon; https://redgar598.shinyapps.io/BECon/) provided in previous studies [39, 40].

### GMV, DMPs, clinical information correlation analysis

We also test whether GMV changes and DMPs components were associated with clinical symptoms in patients with MDD. Using the SMLR regression model, we examined the relationship between GMV alterations, DMPs, and clinical measures such as the Hamilton Depression Rating Scale (HAMD) total scores and Hamilton Anxiety Rating Scale (HAMA) total scores in patients with MDD. The significant level was set at *p* < 0.05.

## Results

### GMV abnormalities and GMV spatial expression associated genes in MDD

The schematic overview of the workflow in this study is shown in Fig. 1. The detailed demographic and clinical data of the participants are summarized in Table S1. We first found patients with MDD had significantly decreased GMV in left hemisphere frontal cortex regions, mainly in the inferior frontal cortex (IFG), dorsolateral prefrontal cortex (DLPFC), anterior cingulate cortex (ACC), and visual cortex regions in the fusiform face complex region (FFC) and posterior inferotemporal region (PIT) (*p* < 0.05, FDR corrected, Table S2, Fig. S1). The GMV of these regions was shown to decrease in patients (*p* < 0.05) when considering medication effects as a covariate (Table S3). The GMV changes of these regions in the right hemisphere were also presented in Supplementary Table S4.

**Fig. 2:**
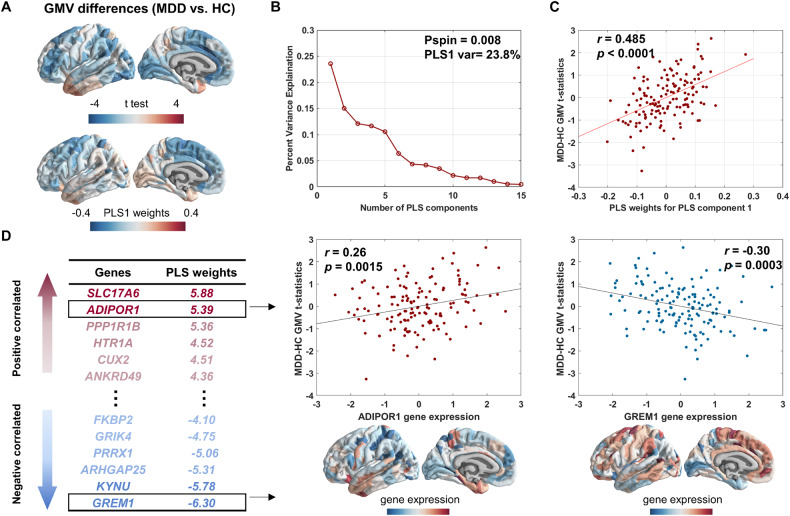
Imaging transcriptomics analysis of abnormal GMV in MDD. **a** Changes in regional GMV in the left hemisphere form *t*-test between MDD and HC (upper) and A weighted gene expression map of regional PLS1 scores in the left hemisphere(lower). **b** A descending curve of variance explanation ratios in each component from PLS regions mode. The PLS1 had the highest variance explanation ratio, 23.8, and the weights of PLS1 were correlated with GMV by spatial correction (Pspin = 0.008). **c** A scatterplot of regional PLS1 scores (a weighted sum of 10,027 gene expression scores) and regional changes in GMV (Pearson’s *r* = 0.485, *p* < 0.0001). **d** Ranked PLS1 loadings GMV-related genes positively (i.e., ADIPOR1: Pearson’s *r* = 0.26, *p* = 0.0015; GREM1: Pearson’s *r* = −0.30, *p* = 0.0003).

We then used PLS regression to determine differences between regional GMV in the left hemisphere and gene expressions. The first component (PLS1) is defined as the spatial map that captures the greatest fraction of total gene expression variance across cortical areas. The PLS1, with explained 23.8% of the variance, showed spatial correlations with GMV changes (Pspin = 0.008, this permutation test randomly “spins” the GMV map to account for spatial correlation, Fig. 2A, Fig. 2B). We found that the PLS1 weighted gene expression map was spatially correlated with the case–control t-map (Pearson’s *r* = 0.48, *p* < 0.0001, Fig. 2C). We ranked genes by the normalized weights of PLS1 based on permutation tests and found 871 PLS1+ (*Z* > 3) and 561 PLS1− (*Z* < −3) (all *p* < 0.05 FDR corrected) positively (or negatively) weighted gene expressions were overexpressed (or under-expressed) as increased (or decreased) regional changes in GMV, respectively. The Top PLS genes associated with GMV changes are shown in Fig. 2D.

### The overlap of DMPs genes and PLS genes

**Fig. 3:**
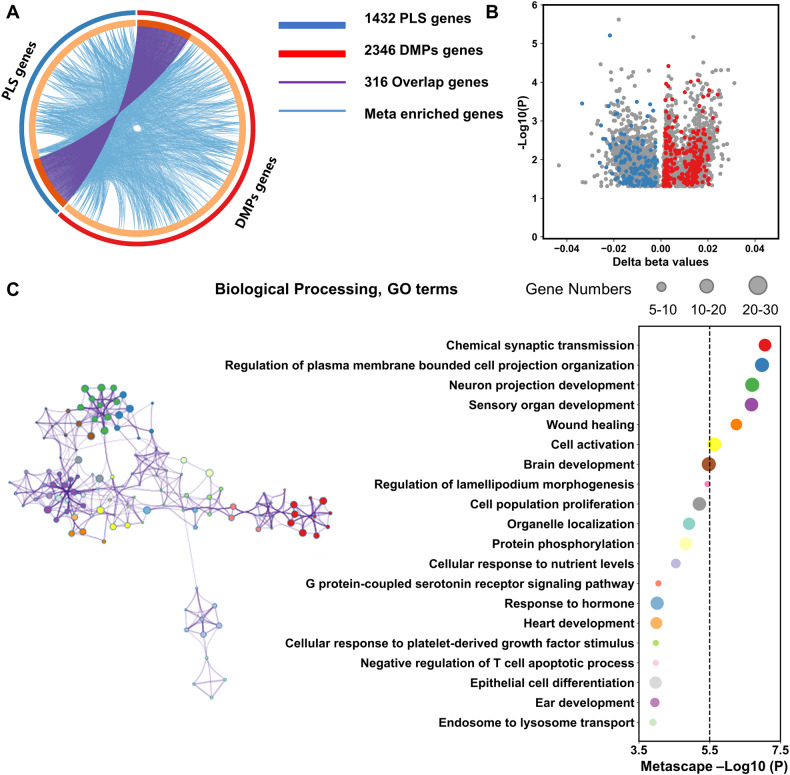
Pathway meta enrichment of Aberrant DM genes and PLS genes. **a** There were 316 overlap genes (purple lines) between 2346 DMPs genes (red loop) and 1432 PLS genes (blue loop). **b** The delta methylation beta values of all the DMPs were showed: gray dots: DMPs not in PLS genes; blue dots: abnormal DMPs (MDD < HC) in overlap genes; red dots: abnormal DMPs (MDD > HC) in overlap genes. **c** The pathways were enriched by the 316 overlap genes.

In total, 316 genes constituted the overlapped genes between 1432 PLS gene and 2346 DMPs genes (Fig. 3A). The delta beta values of DMPs in the overlapped genes were shown in Fig. 3B. The overlapped genes were significantly enriched in the biological processing mainly involving in the neurodevelopmental, neurotransmitter, cellular response to stimulus and metabolic process, most of which were consistent with the pathways enriched by only PLS genes and by meta enrichment analysis (Figs. 3C, S2, S3 and Table S5, S6).

### DNA methylation and GMV associations in MDD

**Fig. 4:**
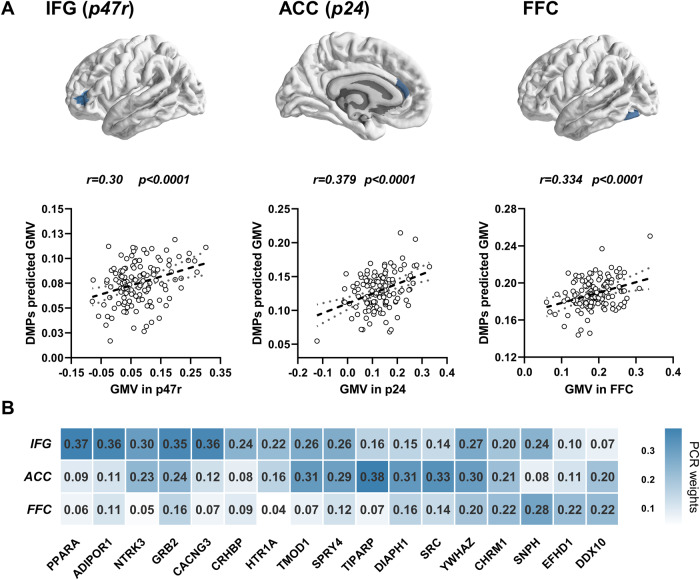
Abnormal methylated genes associated with regional GMV changes in MDD. **a** The associations between components of altered methylated genes and GMV changes in MDD, including component 2 and 22 are correlated with GMV in IFG (p47r) (regression *F* = 5.97, *p* = 0.03 FDR corrected, predicted value correlation *r* = 0.30, *p* < 0.0001), component 12, 17, 20, and 23 are correlated with GMV in ACC (p24) (regression *F* = 5.22, *p* = 0.006 FDR corrected, predicted value correlation *r* = 0.379, *p* < 0.0001), component 7, 9, and 25 are correlated with GMV in FFC (regression *F* = 4.65, *p* = 0.04 FDR corrected, predicted value correlation *r* = 0.334, *p* < 0.0001). **b** The top DMPs genes with the highest sum of weights in components associated with each of the regions were summarized. The distinct pattern of epigenetic changes associated with GMV was shown.

We used PCA dimensionality reduction for those DMPs in the overlap genes and finally got 25 components (Comp) with an accumulated explained variance of 80.1% (Fig. S4). All of the 25 components were used in SMLR models to predict GMV in patients. The regression model predicted GMV values were significantly positively related to the true GMV values in FFC, IFG (p47r), and ACC (p24) regions (Fig. 4A, Table S7). Moreover, the top 25 DMPs components were selected as features to predict GMV individually. LOOCV was performed, and Pearson correlation coefficients results showed significant correspondences between real GMV and predicted GMV values in IFG (*r* = 0.22, *p* = 0.012), ACC (*r* = 0.27, *p* = 0.0012), FFC (*r* = 0.1854, *p* = 0.035) across individual patients with MDD (Fig. S5). We found 17 DMPs with the Top weights (weights > 0.2) in PCA components, which were significantly correlated with GMV in IFG, ACC, and FFC. The 17 DMPs and their annotated genes are present in Fig. 4B and Table S8. These associations between DMPs and GMV showed the region-specific patterns among IFG, ACC, and FFC. The DMPs in genes, including PPARA, ADIPOR1, NTRK3, GRB2, CACNG3, CRHBP, and HTR1A, had higher weights that are correlated with GMV in IFG. Meanwhile, the DMPs in genes, including TMOD1, SPRY4, TIPARP, DIAPH1, SRC, YWHAZ, and CHRM1, had higher weights that are correlated with GMV in ACC. While the DMPs in genes including CHRM1, SNPH, EFHD1, and DDX10 had higher weights that are correlated with FFC (Fig. 4B).

**Fig. 5:**
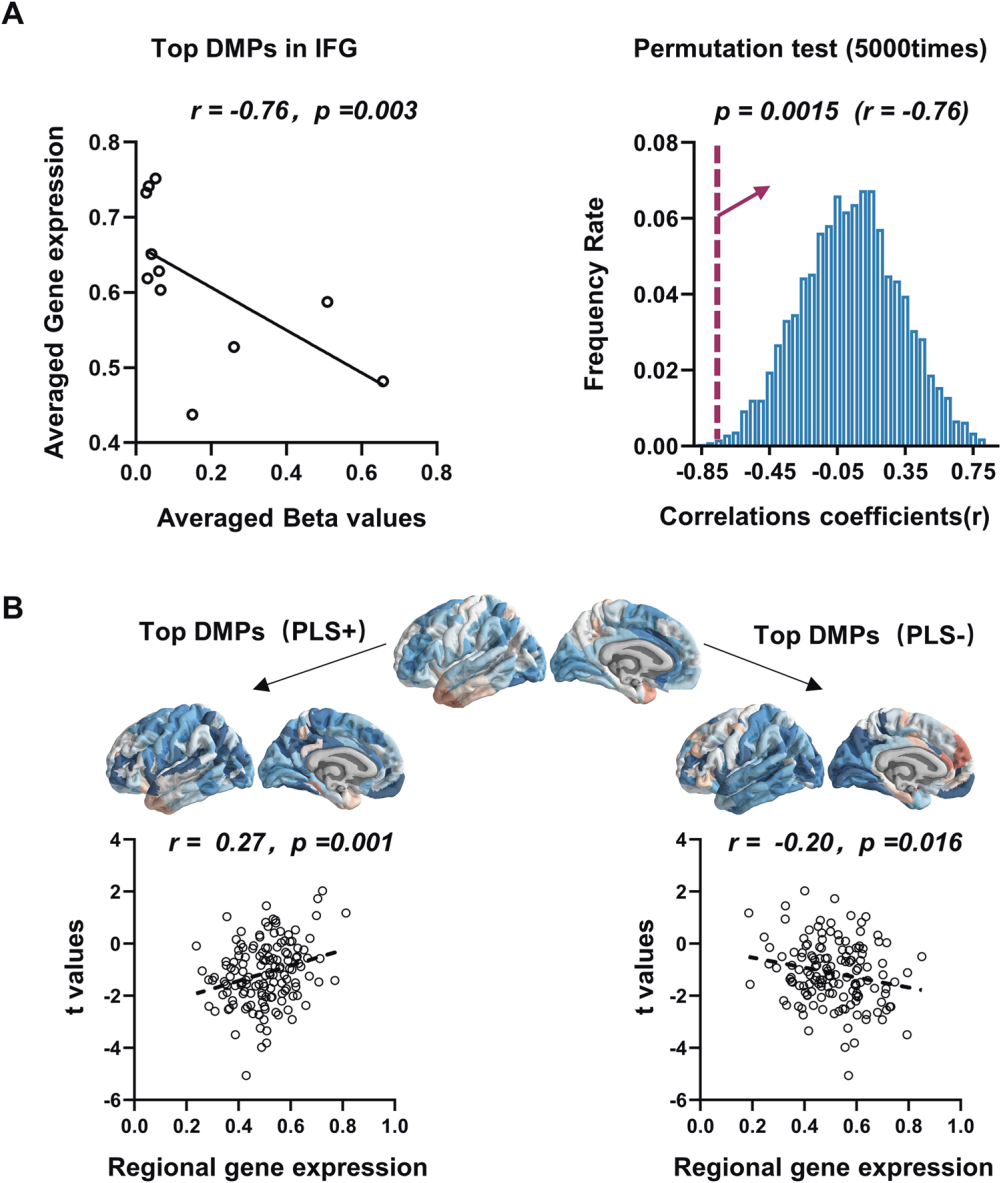
Associations of Top weighted DMPs methylation and gene expression in IFG. **a** The averaged methylation beta values of the top DMPs with higher weights in components associated with GMV in IFG were found to be negatively correlated with their averaged gene expressions in IFG. **b** The 5000 times permutation test was performed to show the correlation coefficients between randomized DMPs methylation and gene expressions in IFG. The distributions of the permutations were present as blue bars. The one-way cumulative probability was set permutation *p* value (*p* = 0.0015) and shown in red color. **c** The gene expressions mapping (left brain map) of top DMPs genes with PLS+ (PLS *z* values > 0) were positively correlated with t maps (middle brain map) of GMV changes in MDD (*r* = 0.27, *p* = 0.001); the gene expressions mapping(right brain map) of top DMPs genes with PLS− (PLS *z* values < 0) were negatively correlated with t maps (middle brain map) of GMV changes in MDD (*r* = −0.20, *p* = 0.016).

Moreover, we found the methylation status of 11 top weights DMPs was significantly negatively correlated with gene expression in the IFG region (*r* = −0.76, *p* = 0.003, permutation test *p* = 0.0015, Fig. 5A). We also extracted the regional gene expression of the 11 Top DMPs genes and found that 8 PLS+ genes were significant positively correlated with GMV changes in MDD (*r* = 0.27, *p* = 0.001) and 3 PLS− genes were significant negatively correlated with GMV changes *t* values (*r* = −0.20, *p* = 0.016) (Fig. 5B). The consistent hypomethylated beta values of the Top DMPs in blood and PFC region were validated (Table S10). The details are presented in Supplementary Table S9, Figs. S6, S7, and S8.

### GMV and DNA methylation changes correlated with symptoms of patients

We found a statistically significant model for predicting HAMD scores using methylation features (*F* = 6.255, *p* = 0.0026) but found no significant model for predicting HAMD scores using GMV features or relating HAMA scores with either GMV or DMPs features.

## Discussion

In this study, we investigated the genetic underpinnings of MDD-related GMV changes by integrating multiple omics data sources, including transcriptome and DNA methylation data. We observed that significantly decreased GMV were spatially associated with gene expression mainly involved in neural development, neurotransmitter function, and metabolic processes. Moreover, the DMPs were significantly correlated with regional GMV changes within individual patients. Furthermore, we observed a strong negative correlation between the DNA methylation status of DMPs associated with GMV changes and gene expression in IFG. These findings reveal complex associations among transcriptomic features, epigenetic changes, and regional-specific structural variations in MDD and expand our understanding of the genetic mechanisms underlying brain structural deficits in MDD.

Our findings showed that patients with MDD had decreased GMV in ACC, IFG, FFC, PIT, and DLPFC. The findings of GMV abnormalities in prefrontal regions (DLPFC, ACC, and IFG) were in line with those detected in the previous meta-analysis, which summarized the most robust GMV reductions in MDD [3]. It is notable that we validated all of these altered GMV while considering medication effects as covariates. We used AHBA gene expression profiles and the multivariate PLS analysis, to identify genetic correlates of global structural variations in MDD and found that genes associated with GMV changes in MDD are mainly involved in neurodevelopmental, cellular, and metabolic processes. The enriched biological pathways were mostly consistent with previous transcriptional findings based on a meta-analysis of GMV changes [28]. Neurodevelopment-related processes, such as neuron projection development, head development, regulation of nervous system development, and regulation of anatomical structure size, were implicated in the pathogenesis of MDD. Previous GWAS studies also observed that neuron projection development and neuron projection terms were enriched by MDD-risk genes [8, 41]. Regulation of nervous system development and head development were also found to be related to the neuronal cell body in imaging transcriptional studies of MDD [28].

To our knowledge, this is the largest dataset combining MRI and epigenome-wide DNA methylation study. We used high-throughput DNA methylation microarray data and performed a data-driven analysis to identify epigenetic features associated with GMV changes in MDD. The imaging transcriptomic analysis was used as the feature selection strategy to identify brain-wide GMV change-related genes and further reduce the high dimensions of the candidate CpG sites. The imaging transcriptional analysis is a versatile method for feature selection in our previous imaging genetic studies [42, 43]. In addition, the PCR method could examine both common and region-specific DMPs associated with GMV changes. Besides, we also found that enriched biological processes of genes with DMPs and those detected in the imaging-transcriptomic spatial correlations overlapped in neurodevelopment, response to stimulus, and energy metabolic processes. The associations between methylation of DMPs and GMV changes across individuals could also serve as a validation for genetic features identified through spatial imaging-transcriptomic regression across different brain regions. Thus, the proposed analysis pipeline in this study may prove to be valuable in revealing associations among GMV changes, gene expression, and DNA methylation abnormalities in MDD. In this study, we also observed that the DNA methylation status of DMPs was significantly associated with patients’ HAMD total scores. These findings suggest that epigenetic changes in blood, which were related to brain structural deficits, may serve as a potential biomarker for predicting clinical symptoms in MDD patients.

In our study, we identified region-specific epigenetic features associated with GMV changes. Concretely, genes including PPARA, ADIPOR1, NTRK3, GRB2, CACNG3, CRHBP, and HTR1A, which are involved in metabolism, neurotransmitter and synaptic plasticity, stress response, were strongly related to GMV in IFG. The polymorphisms of most of these genes had been previously reported to be associated with the risk of MDD [8, 44, 45]. Previous findings indicated that adiponectin acts on 5-HT neurons through ADIPOR1 receptors to regulate depression-related behaviors in a sex-dependent manner [46]. Low plasma adiponectin levels are associated with insulin resistance and can increase the risk of depression and anxiety. Some clinical studies indicate a negative correlation between depression severity and circulating adiponectin. The ADIPOR1 gene encodes a protein that acts as a receptor for adiponectin, a hormone secreted by adipocytes that regulates fatty acid catabolism and glucose levels. Adiponectin is a recently described adipokine that has been recognized as a key regulator of insulin sensitivity and tissue inflammation. The abnormal DNA methylation status of the promoter region in the ADIPOR1 gene may affect its gene expression and then might have effects on the protein related to adipokine. Our finding also provided that the disturbances in adipokine secretion can be an independent risk factor for depression, as previous results [46, 47]. Besides that, a previous study implicated that chronic stress significantly decreased the mRNA of PPARA in mice models, and The PPARA agonist WY14643 improved depressive-like behavior, which suggested PPARA is a therapeutic target for depression [48, 49]. PPAR family genes that regulate stress response play a role in several neural psychopathologies by mediating anti-inflammatory and metabolic actions and directly regulating synaptic transmission and the propagation of nerve signals. Our findings also extend our understanding of the molecular role of the epigenetic changes of the PPARA gene and its gene expression pattern, which were associated with brain structural abnormality in the PFC region in MDD. Moreover, CHRM1 and NTRK3, which are involved in neurodevelopment and synaptic signaling functions, were found to be associated with GMV changes in IFG and ACC. In general, our finding suggested that the genes involved in neurotransmitter and synaptic plasticity might had a common effect on brain structural changes, and both epigenetic changes and transcriptional values of the stress-related genes play an important role in GMV reduction in the prefrontal cortex.

There are several limitations in this study that need to be addressed. Firstly, the statistical power of DNA methylation microarray analysis was limited due to the small sample size used for identifying DMPs. We mitigated this by selecting DMPs that overlapped with candidate PLS genes and controlling for potential false positive rates in the multiple tests conducted to examine the associations between DMPs and changes in GMV in patients. Future studies with larger sample sizes, including multi-ethnic cohorts, would be desirable to improve the statistical power and generalizability of the findings. Secondly, the examination of transcriptome-neuroimaging relationships across different groups was limited by the use of gene expression profiles from only six postmortem healthy human brains in the AHBA. Additionally, the AHBA dataset included data for the right hemisphere of the brain for only two participants, which restricts the representation of the entire brain in relation to the transcriptional changes and MDD-related GMV alterations. Consequently, we did not attempt to analyze the associations between epigenetic markers, gene expression, and GMV changes specifically in the right hemisphere. Future studies should aim to incorporate larger and more diverse datasets to explore these relationships comprehensively. Thirdly, we included a subset of adolescent participants in our study to increase the sample size. To account for potential confounding effects, we regressed the influences of age and gender on GMV and DMPs in our statistical analyses. However, the complex effects of medication on GMV and DNA methylation might not have been completely eliminated, even with the inclusion of these covariates. Therefore, it is important to conduct further studies with larger samples of drug-naïve MDD patients to better understand the associations between these variables while minimizing potential confounding factors.

In summary, we utilized an integrative omics approach to investigate the genetic basis of MDD-related GMV changes at the level of transcriptional and epigenetic regulation. By combining transcriptome and DNA methylation data, we were able to identify key genetic determinants underlying region-specific structural variations in MDD. Our findings suggest that GMV abnormalities in MDD may have a transcriptomic and epigenetic basis. This imaging-transcriptomic-epigenetic integrative analysis provides spatial and biological links between the morphological changes of the central nervous system and peripheral molecular changes in MDD.

### Supplementary information

supplemental material

## Data Availability

Human gene expression data that support the findings of this study are available in the Allen Brain Atlas (“Complete normalized microarray datasets”, https://human.brainmap.org/static/download). The probe-to-gene annotations were obtained by the Re-annotator toolkit (v1.0.0, https://sourceforge.net/projects/reannotator/). The data that support the findings of this study are available from the corresponding author through request.

## Code availability

The code for gene expression preprocessing can be found in a previous study and can be downloaded at https://github.com/BMHLab/AHBAprocessing. The code that supports the findings of the present study is available at https://github.com/zhengjunjie1234/MDD_gmv_methylation.git.
