# Supplementary material for: Integrative omics analysis reveals epigenomic and transcriptomic signatures underlying brain structural deficits in major depressive disorder
Source: Transl Psychiatry. 2024 Jan 9;14:17. doi: 10.1038/s41398-023-02724-8 (PMC10776753; doi:10.1038/s41398-023-02724-8)
Supplement: Supplementary file 1 — supplemental material [file 41398_2023_2724_MOESM1_ESM.docx]

**Integrative Omics Analysis Reveals Epigenomic and Transcriptomic Signatures Underlying Brain Structural Deficits in Major Depressive Disorder**

**Supplemental Materials**

**These supplemental materials include:**

**Supplementary Methods.**

**Supplementary Results.**

**Table S1A.** Demographic, clinical characteristics of MDD patients and healthy controls.

**Table S1B.** Demographic, clinical characteristics of participants having both neuroimaging and DNA methylation data.

**Table S2.** GMV abnormalities in MDD in left hemisphere.

**Table S3.** GMV abnormalities in MDD in left hemisphere considering medication as covariate.

**Table S4.** GMV abnormalities in MDD in right hemisphere considering medication as covariate.

**Table S5.** The biological processing pathways enriched by overlapped genes between DMPs genes and PLS genes.

**Table S6.** Meta enriched biological processing pathways DMPs genes and PLS genes.

**Table S7.** DMPs abnormalities in MDD predicted GMV by SMLR model with 25 components.

**Table S8.** Top weighted DMPs CpG sites.

**Table S9.** Top weighted DMPs CpG sites and genes associated with IFG.

**Table S10.** Top weighted DMPs CpG sites beta values in blood and PFC.

**Figure S1.** Significant abnormal GMV in left hemisphere in MDD.

**Figure S2.** The PLS genes enriched biological processing pathways.

**Figure S3.** Heatmap of enriched terms across DMPs genes and PLS genes.

**Figure S4.** The PCA components used in SMLR regression.

**Figure S5.** LOOCV of DMPs components features based prediction for GMV.

**Figure S6.** The gene methylation and expression values of Top weighted DMPs genes associated with IFG.

**Figure S7.** Top 2 weight DMPs correlated with GMV in MDD.

**Figure S8.** Top weighted DMPs CpG sites beta values in blood and brain by BECon Tools.

**Supplementary** **Methods**

**Participants**

All participants were evaluated using the Structured Clinical Interview for the Diagnostic and Statistical Manual of Mental Disorders, 4th Edition, Text Revision (DSM-IV-TR). Participants younger than 18 years were evaluated using the Schedule for Affective Disorders and Schizophrenia for School-Age Children-present and Lifetime Version (K-SADS-PL). All patients were recommended for the group and assessed by two experienced clinical psychiatrists. Depression severity was assessed using the 17-item Hamilton Depression Rating Scale (HAMD-17), while the Hamilton Anxiety Rating Scale (HAMA) was used to evaluate the severity of anxiety symptoms. Patients were excluded if they had epilepsy, a history of alcohol/drug abuse or dependence, head trauma, history of a major physical or nervous system disease, any electroencephalography abnormalities, diabetes, thyroid disease, other medical history, or any MRI contraindications. The HC subjects were recruited by advertisement. The HC subjects did not have a current Axis I disorder; that is, history of psychotic mood, or other Axis I disorders in their first-degree relatives according to a detailed family history, or any abnormalities on brain images. The participants over 18 years old signed a written consent form themselves. If the participants age were less than 18 years, their parental/legal guardian provided written informed consent. This study was approved by the Ethics Committee of the first affiliated Hospital of China Medical University.

**Image acquisition and MRI processing**

Structural MRI scanning was conducted on a Signa HDx 3.0T superconductive MRI system (GE Healthcare, Little Chalfont, UK) at the First Affiliated Hospital of China Medical University, Shenyang, China. Three-dimensional, high-resolution, and T1-weighted images were acquired using a three-dimensional fast spoiled gradient-echo sequence with the following parameters: Repetition time/Echo time, 7.2/3.2 ms; Flip angle, 13°; image matrix, 240 × 240; field of view, 240 × 240 mm2; 176 contiguous 1.0 mm slices with a gap; and voxel size, 1.0 mm3. All of the participants were required to remain in a prostrate position, relax with their eyes closed and stay awake.

T1-weighted images were preprocessed using the Computation Anatomy Toolbox (CAT 12; Christian Gaser; Department of Psychiatry, University of Jena) implemented in Statistical Parametric Mapping (SPM 12; Wellcome Department of Cognitive Neurology, University of London, UK) for voxel-based morphometry (VBM) [1]. Briefly, a fully automated procedure for standard voxel-based morphometry (including spatial registration, tissue segmentation and bias correction of intensity non-uniformities) was conducted, resulting in GMV images. Total intracranial volume (TIV), calculated as the sum of GM, WM, and CSF volumes, was used as a covariate for further statistical analyses. The HCP (https://humanconnectome.org/) atlas partitioned the cortex into 360 cortical regions (180 regions in each hemisphere), was used as the regions of interest (ROI) template [2]. ROI-wise gray matter density for each brain region was used for investigating abnormalities in MDD.

**Imaging transcriptomic analysis using GMV changes and gene expression**

**Preprocessing of Allen Human Brain Atlas (AHBA) Data**

Transcriptional profiles were obtained from the Allen Human Brain Atlas (AHBA) website (<https://human.brain-map.org/>). The gene expression datasets were extracted from the post-mortem brain tissues of six donors. The donors were a 24-year-old (H0351.2001) and a 39-year-old (H0351.2002) African American male; a 57-year-old (H0351.1009), a 31-year-old (H0351.1012) and a 55-year-old (H0351.1016) European-ancestry male; as well as a 49-year-old Hispanic female (H0351.1015). Further details concerning the gene expression data are also provided at Allen Human Brain Atlas platform (<http://www.brain-map.org>). The profiles included 20,737 gene expressions represented by 58,692 probes. As right hemisphere samples were only available in two out of the six donors in the AHBA dataset, only tissue samples from the left hemisphere (n = all 6 donors) were included.

The expression data were preprocessed according to the 5 major steps: (i) verifying probe-to-gene annotations; (ii) filtering probes not exceeding background noise; (iii) selecting representative probes to index expression for each gene; (iv) assigning and mapping samples to the HCP atlas (https://humanconnectome.org/); (v) normalizing expression values to account for outlying values and inter-participant variances. The open-source code concerning the preprocessing procedure was used (<https://github.com/BMHLab/AHBAprocessing>). After preprocessing, each tissue sample had 10,027 probes (Morgan et al., 2019). All brain tissue samples were then spatially coregistered to the HCP atlas, which consists of 360 cortical regions. The mean gene expression value of all brain tissue samples in each region was calculated, and the expression level of all genes in the left hemisphere regions was used for regression analysis.

**Partial Least Squares Regression Analysis**

Using partial least squares (PLS) regression, as previously performed in other imaging transcriptomics studies [3, 4] we examined the spatial associations between the T-statistic map of GMV alterations and gene expression values in HCP Atlas left hemisphere (180 regions). Gene expression data were set as predictor variables, and the T-statistic maps were response variables. We selected the first component in the PLS (PLS1), which was the linear combination of gene expression values that explained the most variance in associations with the T-statistic maps, for further analyses. To test the null hypothesis that PLS1 explained no more covariance between the brain-wide expression scores and T-statistic maps than expected by chance, we used a permutation test (5000 times) based on spherical rotations, accounting for spatial autocorrelation.[5] We calculated the Pspin value from the permutation test. We used a Pearson linear correlation test to examine the linear relationship between PLS1 weights and T-statistic maps. To evaluate the statistical significance of genes in PLS1, we used bootstrapping to calculate the weights of PLS1 genes. We calculated the ratio of the weight of each regional gene expression to its bootstrap standard error and transformed it into Z-values. The P values were calculated from bootstrapping permutation. After FDR correction (p < 0.05), we obtained the gene set that reliably contributed to PLS1. Then we selected PLS1 genes with weights |Z| > 3, p < 0.05 corrected as candidate genes whose spatial expression correlated with GMV abnormalities in MDD.

**Principal Component Regression between individual GMV and DNA methylation**

**DNA Methylation preprocessing**

The Infinium Human Methylation850 (850K) microarray was used to assess DNA methylation levels. To avoid any batch effects, the samples were processed in a random order. All samples passed the quality assessment for assay performance requirements that were implemented in the Genome Studio software integrated controls dashboard. The DNA methylation levels were expressed as β-values, ranging from 0 to 1, and were calculated as M/(M+U), where M represents the signal from methylated beads and U represents the signal from unmethylated beads at the targeted CpG site. The β-values were corrected for batch and positional effect and cell heterogeneity using the R package ChAMP[6]. The genome coordinates provided by Illumina (GRCh37/hg19) were used to map the independent methylation CpG sites to specific genes. For statistical analysis, CpG sites located within the promoter regions of genes were extracted to examine the regulation of DNA methylation on gene expression.

**Overlapping transcriptomic and epigenomic features**

For statistical analysis, CpG sites located within the promoter regions of genes were extracted to examine the regulation of DNA methylation on gene expression. First, we performed a general linear model (GLM) and t test for each CpG site with age, gender, education, and medication as covariates, to identify differentially methylated CpG positions (DMPs) in MDD compared to HC. The DMPs were selected at significant levels of p < 0.05 uncorrected. All of genes with significant DMPs were designated as DMPs genes. Then, we performed biological processing gene enrichment analysis for PLS genes and the overlapped genes between PLS genes and DMPs genes using an online tool Metascape (<https://metascape.org/>). We also performed the meta enrichment analysis for both PLS1 and DMPs genes. The biological processing genes were used the annotations in Gene Ontology database(<https://geneontology.org/>). The top 20 enriched pathways with significant p < 0.05 FDR corrected are summarized. Finally, we selected the DMPs from the genes overlapped between PLS1 genes and DMPs genes which were significantly enriched in the pathways as features, and extracted individual patients DMPs methylation status beta values to analyze the correlations between DNA methylation and GMV changes.

**The Predict model of DMPs features on GMV in patients with MDD**

We also performed leave one out cross validation (LOOCV) to validate DMPs features prediction for GMV in patients with MDD. For each cross validation, we left one sample out as test sample, and selected DMPs components as features and trained multiple linear regression model to predict GMV of the altered regions using the other samples as training samples. Then all the sample will have predicted GMV values. Finally, we used Pearson correlation coefficients to characterize corresponds between the real GMV and predicted GMV values in all patients with MDD. This LOOCV was used to validate the finding of the associations between GMV and DMPs components using SMLR models for each altered region within MDD group.

**The associations between DMPs methylated status and DMPs gene expression**

We also tested the DNA methylation regulation effects on gene expression. We firstly calculated the Pearson Correlation Coefficients (r values) between DMPs beta values and DMPs components which were significantly correlated with GMV by PCR modeling. The averaged absolute r values of the DMPs were set as weights of DMPs features. The weights of the DMPs features in the correlated components were ranked and their annotated genes were summarized for each significant regression model. The Top 10 weighted DMPs (weights >0.2 and significantly correlated with DMPs components (p<0.05)) in all patients of MDD were extracted. We used Spearman linear correlation to calculate the associations between DMPs methylated states and gene expression of their annotated genes in each GMV changed regions. The permutation test was set 5000 times with randomized DMPs to present the significance. The significant level of permutation test was set p < 0.05, FDR corrected. We also validated the consistent methylation status between blood and brain in the Top DMPs using online tools (Blood Brain DNA Methylation Comparison Tool, https://epigenetics.essex.ac.uk/bloodbrain/) and Blood–brain Epigenetic Condordance; BECon; https://redgar598.shinyapps.io/BECon/) provided in previous studies [7, 8].

### Supplementary Results

**Demographics and clinical behaviors**

The detailed demographic and clinical data of the participants are summarized in **Table S1 and Table S1B**. The 269 individuals with MDD (60 adolescents and 209 adults) and 458 healthy participants (30 adolescents and 420 adults) were recruited in our study. There was no significant difference in age and gender between MDD and HC groups. 129 MDD patients and 179 HC were collected to had venous blood DNA extraction pursuant used in DNA methylation analysis.

**GMV Spatial Expression Associated Genes in MDD**

We used the AHBA, a whole brain transcriptomic dataset, to obtain brain wide gene expressions. Only the left hemisphere was considered in our analysis. As a result, a matrix (180 regions × 10027 gene expression levels) of transcriptional level values was obtained. After PLS regression analysis, the first component (PLS1) is defined as the spatial map that captures the greatest fraction of total gene expression variance across cortical areas. These GMV changes related genes were significantly enriched in biological process including neurodevelopment (e.g., neuron project development, head development, regulation of nervous system development), neurotransmitter (e.g., synaptic signaling, modulation of chemical synaptic transmission, regulation of ion transport) and response to stimulus (e.g., response to hormone, response to extracellular stimulus) (p < 0.05 FDR corrected) **(Figure S2)**.

Then, we tested whether DMPs genes and GMV spatial expression associated genes overlapped with or were involved in the common biological process in MDD. We first identified 2346 DMPs genes that had significant abnormal methylated CpG sites (p < 0.05 uncorrected). There were 316 genes overlapped in both 2346 DMPs genes and 1432 PLS1 genes (**Figure 3A**). We performed a multi-gene-list meta-analysis using the union of 1432 PLS1 genes and 2346 DMPs genes by aligning the gene ontology (GO) biological processes pathways. After correcting for enrichment terms (p < 0.05 FDR corrected) and discarding discrete enrichment clusters, we found genes functionally enriched in neurodevelopment (e.g., neuron project development, brain development, regulation of nervous system development), neurotransmitter (e.g., synaptic signaling, chemical synaptic transmission, modulation of chemical synaptic transmission), cellular response to stimulus and metabolic process (e.g., negative regulation of protein modification process, response to hormone, protein phosphorylation, G protein-coupled serotonin receptor signaling pathway) biological processes (**Figure S3, Table S6**). These findings suggested that the overlapped genes that had abnormal DNA methylated CpG sites and had spatial expression associated GMV changes in MDD, were involved in the neurodevelopmental, neurotransmitter, cellular response to stimulus and metabolic process.

**DNA methylation abnormalities and GMV prediction**

All of the 25 components were used in SMLR models to predict GMV in patient group. Stepwise regression results showed that GMV in FFC were correlated with methylation Comp 7, Comp 9, Comp 25 (F = 4.65, p = 0.04 FDR corrected); Area p32 prime region in ACC were correlated with methylation Comp 12, Comp 17, Comp 20 and Comp 23 (F = 5.22, p = 0.006 FDR corrected); and IFG were correlated to Comp 2 and Comp 22 (F = 5.97, p = 0.03 FDR corrected) (**Table S7**). The regression model predicted GMV values were significantly positive related to the true GMV values (**Figure 4A**). Moreover, the top 25 DMPs components were selected as features to predict GMV individually. LOOCV was performed and Pearson correlation coefficients results showed significant correspondences between real GMV and predicted GMV values in IFG (r = 0.22, p = 0.012), ACC (r = 0.27, p = 0.0012), FFC (r = 0.1854, p = 0.035) across individual patients with MDD **(Figure S5)**. We found 17 DMPs with the Top weights in PCA components which were significantly correlated with GMV in IFG, ACC, FFC. The 17 DMPs and their annotated genes were present in **Table S8 and Figure 4B**. The GMV correlation weights of genes (e.g., PPARA, ADIPOR1, NTRK3), involving in metabolic process pathways, and MDD-related genes (e.g., CRHBP and HTR1A) involving in neurotransmitter process associated with GMV of IFG comparing to FFC and ACC; and higher weights of genes (e.g., TIPARP, DIAPH1) which in neurotransmitter process pathways for prediction in GMV of ACC; the gene related to neurodevelopmental process (e.g., CHRM1, EFHD1) had higher weights for prediction in GMV of FFC. (**Figure 4B**). We also found the altered DNA methylation of PPARA and ADIPOR1 were significantly correlated with GMV changes in MDD in isolation (p<0.05, FDR corrected, **Figure S7**).

Moreover, we found the methylation status of Top weights (weights > 0.2, p<0.05) DMPs genes were significantly negatively correlated with gene expression in IFG region (r = -0.76, p = 0.003, permutation test p = 0.0015, Figure 5A, 5B). we did not find significant correlations between gene methylation and gene expression in ACC and FFC. These findings suggest methylation regulated gene expression in IFG, which present the regional specific effects.

**Supplementary Tables and Figures**

### Table S1A. Demographic, clinical characteristics of MDD patients and healthy controls.

|  | MDD  (n=269) | HC  (n=458) | *T*/ *X^2^* values | P values |
| --- | --- | --- | --- | --- |
| Demographic characteristics |  |  |  |  |
| Age (mean±sd) | 27.3±10.1 | 28.5±9.5 | 1.48 | 0.13 |
| Gender (male/female) | 75/194 | 170/246 | 1.34 | 0.5 |
| Education (mean±sd) | 12.3±3.5 | 14.3±3.8 | 6.88 | 0.001 |
| Handedness (R/L/Mix) | 239/14/6 | 425/24/3 | 3.61 | 0.16 |
| Clinical characteristics |  |  |  |  |
| First episode, yes | 244(91%) | NA |  |  |
| Illness Duration (months) | 16.8±38.1 | NA |  |  |
| Medication, yes/no | 149/120 | NA |  |  |
| DNA methylation subjects | 129 | 176 |  |  |
| HAMD | 21.0±8.1 |  |  |  |
| HAMA | 18.8±9.4 |  |  |  |

Note: Data were presented as either n (%) or mean ± sd. HC, Healthy Controls; HAMD, Hamilton Depression Scale; HAMA, Hamilton anxiety Scale; R, right handedness; L, left handedness; Mix, mix handedness.

### Table S1B. Demographic, clinical characteristics of participants having both neuroimaging and DNA methylation data.

|  | MDD  (n=129) | HC  (n=172) | *T*/ *X^2^* values | P values |
| --- | --- | --- | --- | --- |
| Demographic characteristics |  |  |  |  |
| Age (mean±sd) | 26.2±9.9 | 27.6±8.5 | 1.35 | 0.17 |
| Gender (male/female) | 38/91 | 68/104 | 3.20 | 0.08 |
| Education (mean±sd) | 12.5±3.5 | 14.6±3.7 | 5.88 | 0.001 |
| Handedness (R/L/Mix) | 117/7/5 | 165/5/2 | 3.72 | 0.15 |
| Clinical characteristics |  |  |  |  |
| First episode, yes | 128(99%) | NA |  |  |
| Illness Duration (months) | 12.5±3.5 | NA |  |  |
| HAMD | 20.1±7.5 |  |  |  |
| HAMA | 18.7±9.2 |  |  |  |

Note: Data were presented as either n (%) or mean ± sd. HC, Healthy Controls; HAMD, Hamilton Depression Scale; HAMA, Hamilton anxiety Scale; R, right handedness; L, left handedness; Mix, mix handedness.

**Table S2. GMV abnormalities in MDD in left hemisphere.**

| Regions | Description  (Glasser 360 atlas) | Cortex | MDD  (mean) | HC  (mean) | T values | P values  corrected |
| --- | --- | --- | --- | --- | --- | --- |
| lh_FFC | Fusiform Face Complex | Ventral Stream Visual Cortex | 0.1920 | 0.2055 | -3.5300 | 0.0114 |
| lh_PIT | Posterior InferoTemporal | Ventral Stream Visual Cortex | 0.1338 | 0.1590 | -5.3543 | 0.0000 |
| lh_p24pr | Area Posterior 24 prime | Anterior Cingulate and Medial Prefrontal Cortex | 0.2014 | 0.2196 | -3.0862 | 0.0394 |
| lh_a24pr | Anterior 24 prime | Anterior Cingulate and Medial Prefrontal Cortex | 0.1361 | 0.1607 | -3.6697 | 0.0079 |
| lh_p32pr | Area p32 prime | Anterior Cingulate and Medial Prefrontal Cortex | 0.1182 | 0.1450 | -4.2060 | 0.0026 |
| lh_s6_8 | Superior6-8 Transitional Area | Dorso Lateral Prefrontal Cortex | 0.1230 | 0.1415 | -3.6658 | 0.0079 |
| lh_25 | Area 25 | Anterior Cingulate and Medial Prefrontal Cortex | 0.0027 | 0.0178 | -3.4293 | 0.0144 |
| lh_p47r | Inferior Frontal Cortex | Orbital and Polar Frontal Cortex | 0.0760 | 0.0950 | -3.0742 | 0.0394 |
| lh_a32pr | Anterior 32 prime | Anterior Cingulate and Medial Prefrontal Cortex | 0.0582 | 0.0869 | -4.0241 | 0.0038 |
| lh_p24 | Area Posterior 24 | Anterior Cingulate and Medial Prefrontal Cortex | 0.1287 | 0.1499 | -3.6955 | 0.0079 |

lh: left hemisphere.

**Table S3. GMV abnormalities in MDD in left hemisphere considering medication as covariate.**

| Regions | Description  (Glasser 360 atlas) | Cortex | MDD  (mean) | HC  (mean) | T values | P values |
| --- | --- | --- | --- | --- | --- | --- |
| lh_FFC | Fusiform Face Complex | Ventral Stream Visual Cortex | 0.1920 | 0.2055 | -2.7959 | **0.0053** |
| lh_PIT | Posterior InferoTemporal | Ventral Stream Visual Cortex | 0.1338 | 0.1590 | -2.7597 | **0.0059** |
| lh_p24pr | Area Posterior 24 prime | Anterior Cingulate and Medial Prefrontal Cortex | 0.2014 | 0.2196 | -1.9889 | **0.0471** |
| lh_a24pr | Anterior 24 prime | Anterior Cingulate and Medial Prefrontal Cortex | 0.1361 | 0.1607 | -3.9044 | **0.0001** |
| lh_p32pr | Area p32 prime | Anterior Cingulate and Medial Prefrontal Cortex | 0.1182 | 0.1450 | -3.5940 | **0.0003** |
| lh_s6_8 | Superior6-8 Transitional Area | Dorso Lateral Prefrontal Cortex | 0.1230 | 0.1415 | -2.7508 | **0.0061** |
| lh_25 | Area 25 | Anterior Cingulate and Medial Prefrontal Cortex | 0.0027 | 0.0178 | -2.0808 | **0.0378** |
| lh_p47r | Inferior Frontal Cortex | Orbital and Polar Frontal Cortex | 0.0760 | 0.0950 | -2.0337 | **0.0423** |
| lh_a32pr | Anterior 32 prime | Anterior Cingulate and Medial Prefrontal Cortex | 0.0582 | 0.0869 | -4.4938 | **0.0000** |
| lh_p24 | Area Posterior 24 | Anterior Cingulate and Medial Prefrontal Cortex | 0.1287 | 0.1499 | -3.5827 | **0.0004** |

lh: left hemisphere.

**Table S4. GMV abnormalities in MDD in right hemisphere considering medication as covariate.**

| Regions | Description  (Glasser 360 atlas) | Cortex | MDD  (mean) | HC  (mean) | T values | P values |
| --- | --- | --- | --- | --- | --- | --- |
| rh_FFC | Fusiform Face Complex | Ventral Stream Visual Cortex | 0.1415 | 0.1524 | -2.439 | 0.0149 |
| rh_PIT | Posterior InferoTemporal | Ventral Stream Visual Cortex | 0.1378 | 0.1506 | -3.399 | 0.0007 |
| rh_p24pr | Area Posterior 24 prime | Anterior Cingulate and Medial Prefrontal Cortex | 0.1666 | 0.1836 | -3.005 | 0.0027 |
| rh_a24pr | Anterior 24 prime | Anterior Cingulate and Medial Prefrontal Cortex | 0.1856 | 0.2056 | -2.649 | 0.0082 |
| rh_p32pr | Area p32 prime | Anterior Cingulate and Medial Prefrontal Cortex | 0.1439 | 0.1570 | -2.341 | 0.0194 |
| rh_s6_8 | Superior6-8 Transitional Area | Dorso Lateral Prefrontal Cortex | 0.07963 | 0.09430 | -2.303 | 0.0215 |
| rh_25 | Area 25 | Anterior Cingulate and Medial Prefrontal Cortex | 0.0489 | 0.0530 | -0.974 | 0.3299 |
| rh_p47r | Inferior Frontal Cortex | Orbital and Polar Frontal Cortex | 0.1383 | 0.1577 | -3.194 | 0.0014 |
| rh_a32pr | Anterior 32 prime | Anterior Cingulate and Medial Prefrontal Cortex | 0.1482 | 0.1655 | -2.858 | 0.0043 |
| rh_p24 | Area Posterior 24 | Anterior Cingulate and Medial Prefrontal Cortex | 0.2465 | 0.2645 | -2.298 | 0.0218 |

rh: right hemisphere.

**Table S5. The biological processing pathways enriched by overlapped genes between DMPs genes and PLS genes.**

| **GO** | **Description** | **Enriched Genes**  **(number)** | **-Log P**  **Corrected** |
| --- | --- | --- | --- |
| GO:0007268 | chemical synaptic transmission | 19 | 3.4 |
| GO:0120035 | regulation of plasma membrane bounded cell projection organization | 24 | 3.4 |
| GO:0031175 | neuron projection development | 24 | 3.4 |
| GO:0007423 | sensory organ development | 22 | 3.4 |
| GO:0042060 | wound healing | 16 | 3.02 |
| GO:0001775 | cell activation | 23 | 2.59 |
| GO:0007420 | brain development | 24 | 2.52 |
| GO:2000392 | regulation of lamellipodium morphogenesis | 4 | 2.51 |
| GO:0008283 | cell population proliferation | 22 | 2.37 |
| GO:0051640 | organelle localization | 18 | 2.16 |
| GO:0006468 | protein phosphorylation | 21 | 2.09 |
| GO:0031669 | cellular response to nutrient levels | 11 | 1.88 |
| GO:0098664 | G protein-coupled serotonin receptor signaling pathway | 4 | 1.5 |
| GO:0009725 | response to hormone | 21 | 1.48 |
| GO:0007507 | heart development | 17 | 1.48 |
| GO:0036120 | cellular response to platelet-derived growth factor stimulus | 4 | 1.48 |
| GO:0070233 | negative regulation of T cell apoptotic process | 4 | 1.48 |
| GO:0030855 | epithelial cell differentiation | 18 | 1.48 |
| GO:0043583 | ear development | 10 | 1.46 |
| GO:0008333 | endosome to lysosome transport | 6 | 1.44 |

**Table S6. Meta enriched biological processing pathways DMPs genes and PLS genes.**

| **GO** | **Description** | **Enriched Genes**  **(number)** | **-Log P**  **Corrected** |
| --- | --- | --- | --- |
| GO:0051129 | negative regulation of cellular component organization | 189 | 56.99 |
| GO:0031175 | neuron projection development | 176 | 53.15 |
| GO:0007610 | behavior | 154 | 44.04 |
| GO:0009725 | response to hormone | 177 | 43.56 |
| GO:0031344 | regulation of cell projection organization | 160 | 43.42 |
| GO:0031400 | negative regulation of protein modification process | 132 | 41.67 |
| GO:0035239 | tube morphogenesis | 154 | 38.14 |
| GO:0007420 | brain development | 166 | 37.07 |
| GO:0030029 | actin filament-based process | 140 | 36.17 |
| GO:0034330 | cell junction organization | 127 | 34.38 |
| GO:0045596 | negative regulation of cell differentiation | 153 | 34.38 |
| GO:0044057 | regulation of system process | 134 | 33.16 |
| GO:0090066 | regulation of anatomical structure size | 122 | 33.15 |
| GO:0007423 | sensory organ development | 131 | 32.54 |
| GO:0051960 | regulation of nervous system development | 115 | 31.35 |
| GO:0050804 | modulation of chemical synaptic transmission | 118 | 31.35 |
| GO:0070848 | response to growth factor | 122 | 31.33 |
| GO:0061024 | membrane organization | 152 | 31.25 |
| GO:0008285 | negative regulation of cell population proliferation | 155 | 31.06 |
| GO:0099536 | synaptic signaling | 112 | 30.56 |

**Table S7. DMPs abnormalities in MDD predicted GMV by SMLR model with 25 components.**

| **Regions** | **Regions Description** | **F values** | **P values** | **P values corrected** |
| --- | --- | --- | --- | --- |
| **lh_FFC** | **Fusiform Face Complex** | **4.6495** | **0.0040** | **0.04** |
| lh_PIT | Posterior InferoTemporal | NaN | NaN | NaN |
| lh_p24pr | Area Posterior 24 prime | 4.9153 | 0.0283 | 0.28 |
| lh_a24pr | Anterior 24 prime | 5.8755 | 0.0167 | 0.16 |
| lh_p32pr | Area p32 prime | NaN | NaN | NaN |
| lh_s6_8 | Superior6-8 Transitional Area | 5.4615 | 0.0210 | 0.21 |
| lh_25 | Area 25 | 4.5069 | 0.0128 | 0.12 |
| **lh_p47r** | **Inferior Frontal Cortex** | **5.9730** | **0.0033** | **0.03** |
| lh_a32pr | Anterior 32 prime | 5.4051 | 0.0055 | 0.05 |
| **lh_p24** | **Area Posterior 24** | **5.2270** | **0.0006** | **0.006** |

**Table S8. Top weighted DMPs CpG sites.**

| **Gene Name** | **CpG sites** | **MDD**  **（mean）** | **HC**  **（mean）** | **T values** | **P values**  **(FDR)** |
| --- | --- | --- | --- | --- | --- |
| EFHD1 | cg17021917 | 0.954 | 0.960 | -2.122 | 0.0453 |
| YWHAZ | cg11839355 | 0.034 | 0.032 | 2.146 | 0.0453 |
| CACNG3 | cg07514158 | 0.061 | 0.066 | -2.762 | 0.0173 |
| CRHBP | cg22323744 | 0.149 | 0.158 | -2.177 | 0.0453 |
| HTR1A | cg11432303 | 0.260 | 0.273 | -2.660 | 0.0200 |
| CHRM1 | cg00848283 | 0.509 | 0.495 | 3.324 | 0.0085 |
| GRB2 | cg24004692 | 0.052 | 0.048 | 1.802 | 0.0726 |
| PPARA | cg24676000 | 0.893 | 0.886 | 2.085 | 0.0460 |
| DDX10 | cg23905010 | 0.241 | 0.226 | 2.815 | 0.0173 |
| SRC | cg24384816 | 0.657 | 0.637 | 2.983 | 0.0131 |
| ADIPOR1 | cg25023057 | 0.041 | 0.038 | 1.884 | 0.0643 |
| DIAPH1 | cg06983483 | 0.024 | 0.021 | 3.722 | 0.0040 |
| SNPH | cg25509338 | 0.090 | 0.084 | 2.288 | 0.0388 |
| TIPARP | cg06068627 | 0.027 | 0.025 | 1.966 | 0.0569 |
| TMOD1 | cg02031942 | 0.030 | 0.028 | 2.369 | 0.0388 |
| NTRK3 | cg21956337 | 0.065 | 0.070 | -2.313 | 0.0388 |
| SPRY4 | cg08265790 | 0.034 | 0.029 | 3.162 | 0.0098 |

FDR: false positive rates correction was performed among these 17 top weights DMPs.

**Table S9.** **Top weighted DMPs CpG sites and genes associated with IFG.**

| CpG sites | Genes | gene expression in IFG | mdd_methylation | hc_methylation | PLS_zvalues |
| --- | --- | --- | --- | --- | --- |
| cg22323744 | CRHBP | 0.437551378 | 0.149403657 | 0.157658534 | -3.349298 |
| cg08265790 | SPRY4 | 0.741627078 | 0.03394267 | 0.029094848 | -3.286862 |
| cg06068627 | TIPARP | 0.732700109 | 0.027312036 | 0.025093283 | -3.12742 |
| cg24004692 | GRB2 | 0.481945692 | 0.656818852 | 0.636879121 | 2.881115 |
| cg24676000 | PPARA | 0.751719576 | 0.051547507 | 0.048010292 | 2.968549 |
| cg21956337 | NTRK3 | 0.603494866 | 0.065256142 | 0.069502376 | 3.161157 |
| cg00848283 | CHRM1 | 0.587360683 | 0.509330731 | 0.494828394 | 3.28144 |
| cg07514158 | CACNG3 | 0.62840247 | 0.061301821 | 0.066346783 | 3.616066 |
| cg02031942 | TMOD1 | 0.618976719 | 0.030205935 | 0.027882797 | 3.6627 |
| cg11432303 | HTR1A | 0.527614387 | 0.260108827 | 0.273155777 | 4.52578 |
| cg25023057 | ADIPOR1 | 0.651162598 | 0.040882631 | 0.038107972 | 5.39842 |

**Table S10. Top weighted DMPs CpG sites beta values in blood and PFC.**

| CpG sites | Genes | beta value in blood  (mean±sd) | beta value in PFC  (mean±sd) | Correlation  r_values  (n=74) | Correlation  p_values  (n=74) |
| --- | --- | --- | --- | --- | --- |
| cg22323744 | CRHBP | No Matched |  |  |  |
| cg08265790 | SPRY4 | 0.125±0.03 | 0.131±0.02 | 0.043 | 0.716 |
| cg06068627 | TIPARP | 0.082±0.01 | 0.085±0.02 | -0.147 | 0.21 |
| cg24004692 | GRB2 | No Matched |  |  |  |
| cg24676000 | PPARA | 0.083±0.03 | 0.085±0.03 | 0.264 | 0.029 |
| cg21956337 | NTRK3 | 0.11±0.03 | 0.095±0.02 | 0.131 | 0.265 |
| cg00848283 | CHRM1 | No Matched |  |  |  |
| cg07514158 | CACNG3 | 0.15±0.03 | 0.10±0.03 | 0.0615 | 0.603 |
| cg02031942 | TMOD1 | No Matched |  |  |  |
| cg11432303 | HTR1A | No Matched |  |  |  |
| cg25023057 | ADIPOR1 | 0.084±0.04 | 0.08±0.02 | 0.025 | 0.828 |

**Figure S1. Significant abnormal GMV in left hemisphere in MDD.**


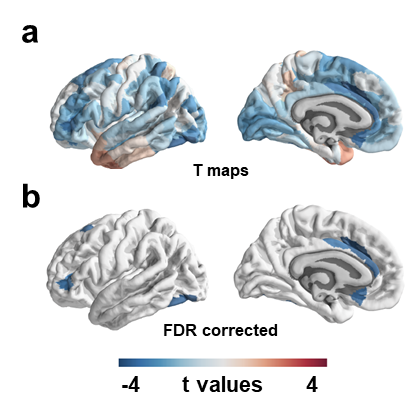


**Figure S1. Significant abnormal GMV in left hemisphere in MDD. a.** the T maps of GMV in patients with MDD comparing to HC. **b.** the significant altered GVM regions of left hemisphere (p <0.05 FDR corrected) were shown.

**Figure S2. The PLS genes enriched biological processing pathways.**


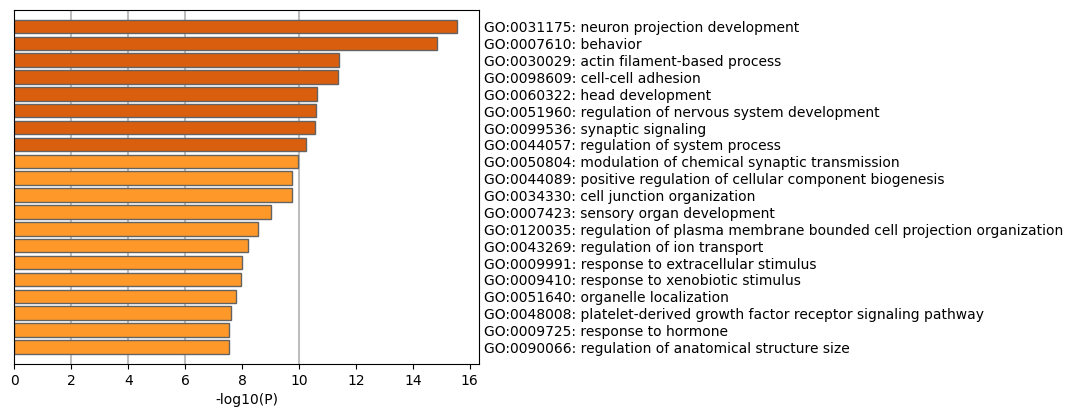


**Figure S2. The PLS genes enriched biological processing pathways.** Top 20 clustered biological processing pathways (p<0.05 FDR corrected) enriched by PLS genes were presented sorted by -logP values.

**Figure S3. Heatmap of enriched terms across DMPs genes and PLS genes.**


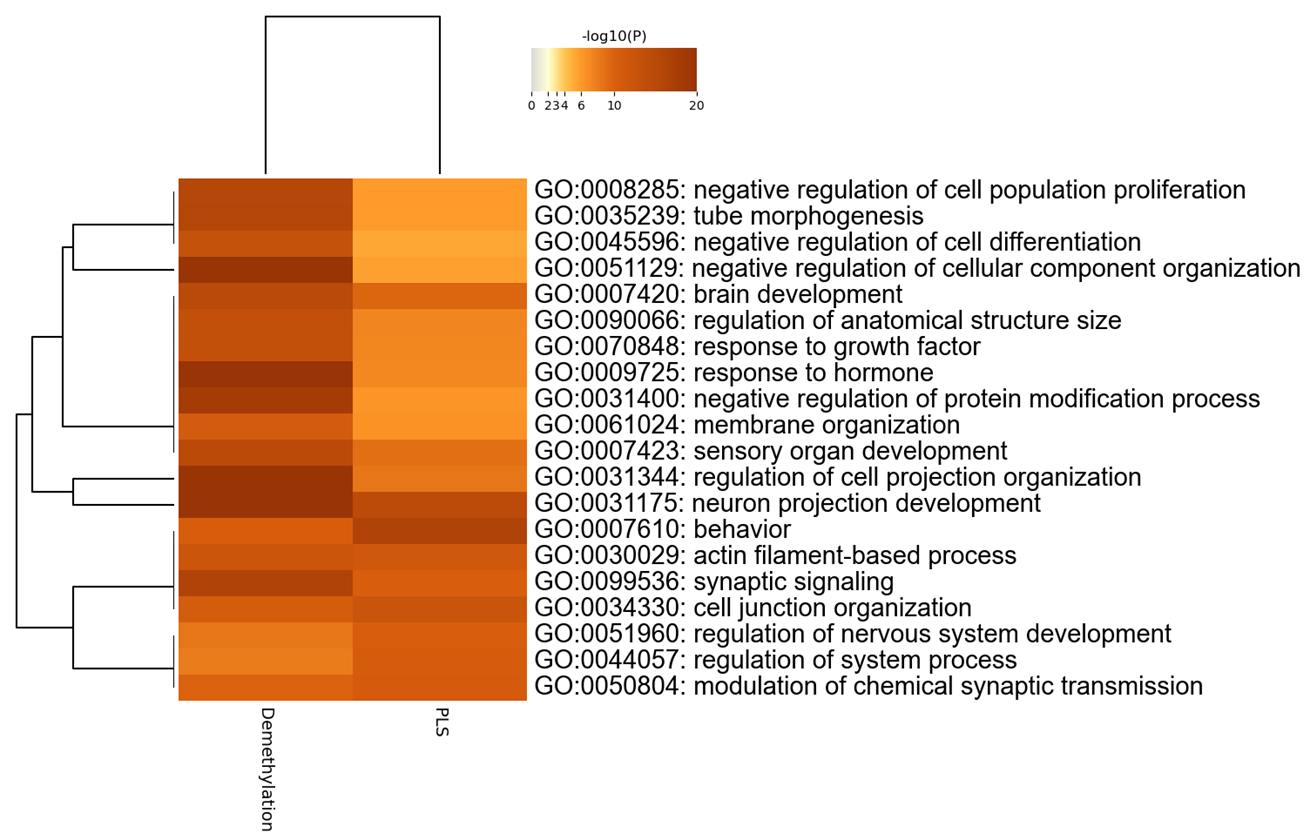


**Figure S3. Heatmap of enriched terms across DMPs genes and PLS genes.** The meta enrichment analysis presented biological processing pathways (p<0.05) enriched by DMPs genes and PLS genes, colored by p-values. Demethylation: DMPs genes, PLS: PLS genes.

**Figure S4. The PCA components used in SMLR regression.**


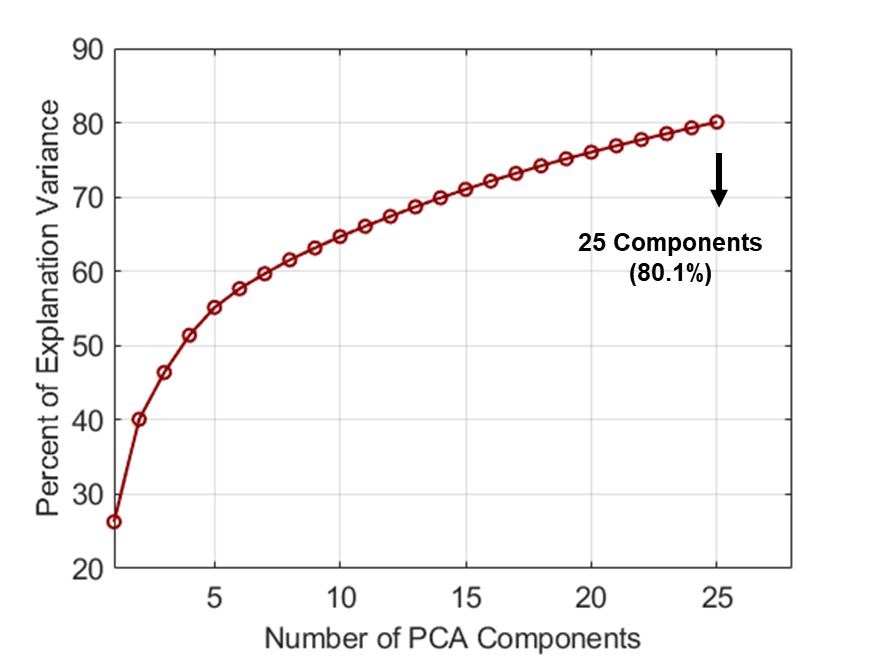


**Figure S4. The PCA components used in SMLR regression.** The points present the accumulated percentages of explanation variance in each number of Principal Component Analysis (PCA) by using abnormal DNA methylation CpG sites of genes which had spatial expression correlation with GMV changes in MDD. 25 components with more than 80% explanation variance were use in SMLR regression model.

**Figure S5. LOOCV of DMPs components features based prediction for GMV.**


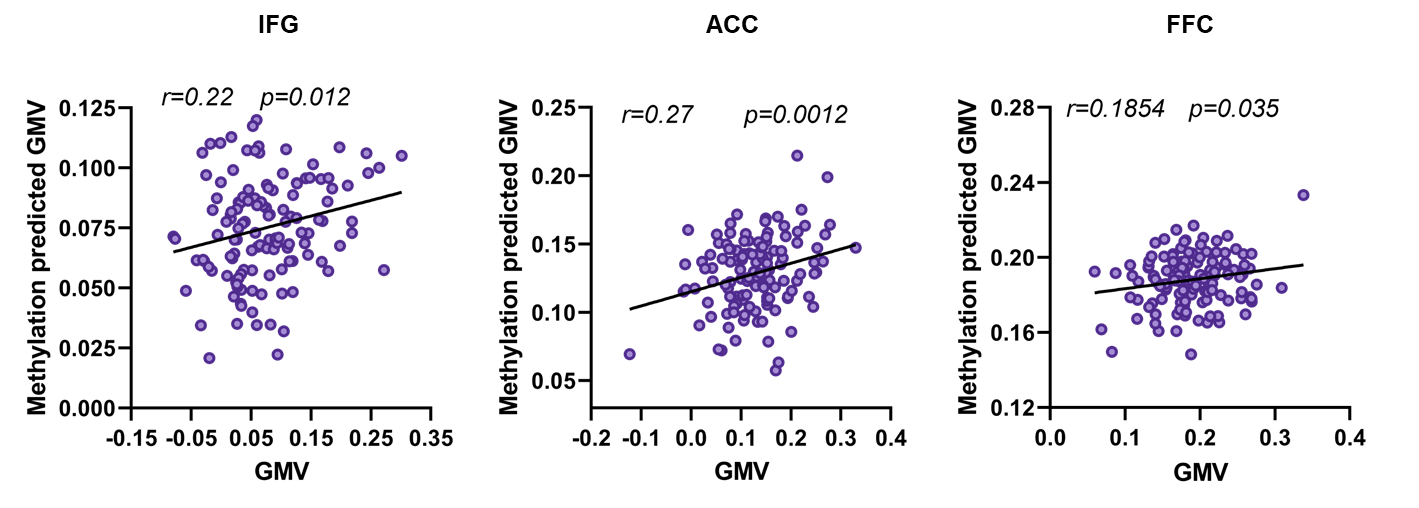


**Figure S5. LOOCV of DMPs components features based prediction for GMV.** The top 25 DMPs components were selected as features to predict GMV individually. LOOCV was performed and Pearson correlation coefficients were calculated between real GMV and predicted GMV values in IFG (r=0.22, p=0.012), ACC (r=0.27, p=0.0012), FFC (r=0.1854, p=0.035) across individual patients with MDD.

**Figure S6. The gene methylation and expression values of Top weighted DMPs genes associated with IFG.**

**
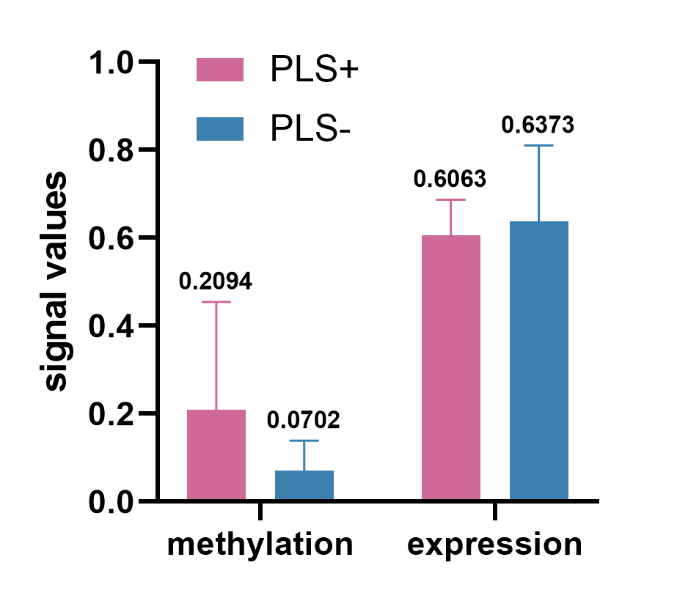
**

**Figure S6. The gene methylation and expression values of Top weighted DMPs genes associated with IFG.** The mean values of methylation beta values and gene expression values were present above the error bar.

**Figure S7. Top 2 weight DMPs correlated with GMV in MDD.**


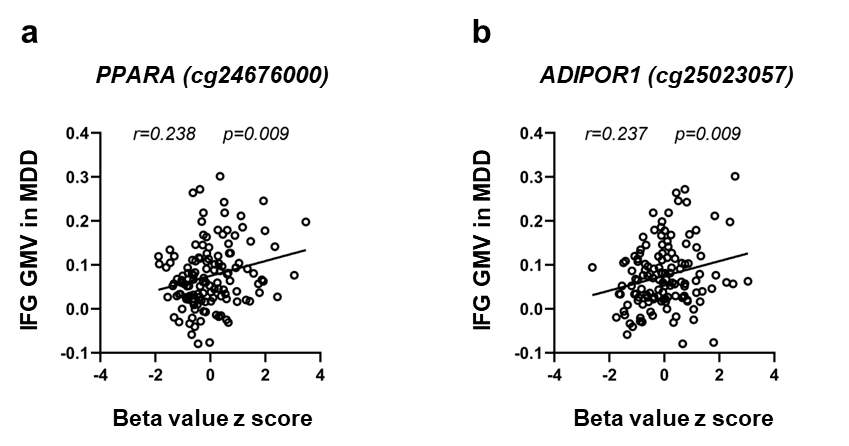


**Figure S7. Top 2 weight DMPs correlated with GMV in MDD.** the top 2 genes CpG site methylations (PPARA,, ADIPOR1) with highest weights in components associated with GMV in IFG were selected. The significant Pearson correlations were found between methylation of each gene (a. PPARA r=0.238, p=0.009, **b.** ADIPOR1 r=0.237, =0.009) and GMV of IFG were calculated.

**Figure S8. Top weighted DMPs CpG sites beta values in blood and brain by BECon Tools.**


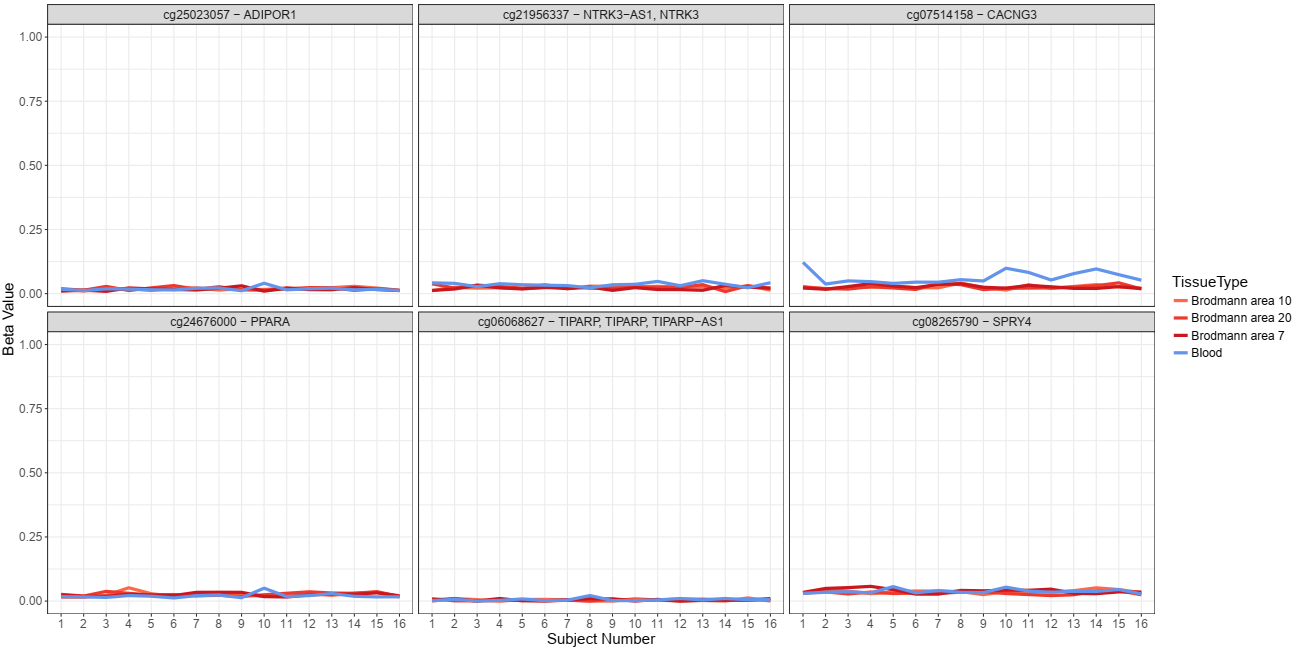


**Figure S8. Top weighted DMPs CpG sites beta values in blood and brain by BECon Tools.** We used BECon (Blood–brain Epigenetic Condordance; <https://redgar598.shinyapps.io/BECon/> ) to validated the consistent methylated status of the Top weighted DMPs cpg sites associated with IFG. The matched cpg sites showed the moderated consistent hypomethylated status between blood and brain regions.

### References

1. Gaser C, Dahnke R, Thompson PM, Kurth F, Luders E. CAT-a computational anatomy toolbox for the analysis of structural MRI data. Preprint at www.biorxiv.org/content/10.1101/2022.06.11.495736v2.full (2022).

2. Glasser MF, Coalson TS, Robinson EC, Hacker CD, Harwell J, Yacoub E *et al.* A multi-modal parcellation of human cerebral cortex. *Nature* 2016; 536(7615)**:** 171-178.

3. Morgan SE, Seidlitz J, Whitaker KJ, Romero-Garcia R, Clifton NE, Scarpazza C *et al.* Cortical patterning of abnormal morphometric similarity in psychosis is associated with brain expression of schizophrenia-related genes. *Proc Natl Acad Sci U S A* 2019; 116(19)**:** 9604-9609.

4. Li J, Seidlitz J, Suckling J, Fan F, Ji G-J, Meng Y *et al.* Cortical structural differences in major depressive disorder correlate with cell type-specific transcriptional signatures. *Nat Commun* 2021; 12(1)**:** 1-14.

5. Romero-Garcia R, Seidlitz J, Whitaker KJ, Morgan SE, Fonagy P, Dolan RJ *et al.* Schizotypy-related magnetization of cortex in healthy adolescence is colocated with expression of schizophrenia-related genes. *Biol Psychiatry* 2020; 88(3)**:** 248-259.

6. Tang L, Liu J, Zhu Y, Duan J, Chen Y, Wei Y *et al.* ANK3 Gene Polymorphism Rs10994336 Influences Executive Functions by Modulating Methylation in Patients With Bipolar Disorder. *Front Neurosci* 2021**:** 755.

7. Edgar RD, Jones MJ, Meaney MJ, Turecki G, Kobor MS. BECon: a tool for interpreting DNA methylation findings from blood in the context of brain. *Transl Psychiatry* 2017; 7(8)**:** e1187-e1187.

8. Hannon E, Lunnon K, Schalkwyk L, Mill J. Interindividual methylomic variation across blood, cortex, and cerebellum: implications for epigenetic studies of neurological and neuropsychiatric phenotypes. *Epigenetics* 2015; 10(11)**:** 1024-1032.
